# A Distributed Image Compression Scheme for Energy Harvesting Wireless Multimedia Sensor Networks

**DOI:** 10.3390/s20030667

**Published:** 2020-01-25

**Authors:** Chong Han, Songtao Zhang, Biao Zhang, Jian Zhou, Lijuan Sun

**Affiliations:** 1 College of Computer, Nanjing University of Posts and Telecommunications, Nanjing 210003, China; B16041718@njupt.edu.cn (S.Z.); B16041621@njupt.edu.cn (B.Z.); zhoujian@njupt.edu.cn (J.Z.); sunlijuan_nupt@163.com (L.S.); 2 Jiangsu High Technology Research Key Laboratory for Wireless Sensor Networks, Nanjing University of Posts and Telecommunications, Nanjing 210003, China

**Keywords:** distributed image compression scheme, solar energy, dynamic adjustment, cluster

## Abstract

As an emerging technology, edge computing will enable traditional sensor networks to be effective and motivate a series of new applications. Meanwhile, limited battery power directly affects the performance and survival time of sensor networks. As an extension application for traditional sensor networks, the energy consumption of Wireless Multimedia Sensor Networks (WMSNs) is more prominent. For the image compression and transmission in WMSNs, consider using solar energy as the replenishment of node energy; a distributed image compression scheme based on solar energy harvesting is proposed. Two level clustering management is adopted. The camera node-normal node cluster enables camera nodes to gather and send collected raw images to the corresponding normal nodes for compression, and the normal node cluster enables the normal nodes to send the compressed images to the corresponding cluster head node. The re-clustering and dynamic adjustment methods for normal nodes are proposed to adjust adaptively the operation mode in the working chain. Simulation results show that the proposed distributed image compression scheme can effectively balance the energy consumption of the network. Compared with the existing image transmission schemes, the proposed scheme can transmit more and higher quality images and ensure the survival of the network.

## 1. Introduction

With the global proliferation of Internet connected devices, efficiency in data transmission and processing is becoming increasingly crucial. Wireless Sensor Networks (WSNs) [1,2] could initially collect the data distributed, and edge computing will enable traditional sensor networks to be effective [3,4,5,6]. Wireless Multimedia Sensor Networks (WMSNs) are a new type of network that adds video, audio, image, and other multimedia information perception functions based on traditional WSNs [7,8]. WMSNs perceive various media information in the surrounding environment through multimedia sensor nodes. This information can be transmitted to the collection nodes via single and multihop relay [9,10]. Collection nodes analyze and process the received data and send the analysis and processing results to the network owner to realize comprehensive and effective environmental monitoring. WMSNs integrate and expand the application of traditional WSNs and are widely used in security and environment monitoring, intelligent transportation and homes, and other applications requiring multimedia information. WMSNs are a typical application motivated by the combination of Wireless Sensor Networks (WSNs) and edge computing [11,12,13,14,15].

Energy consumption is a main research direction for WMSNs. To reduce the energy consumption of network transmission and compression and balance the distribution of network energy consumption, some researchers have proposed the distributed image compression methods [16,17]. Distributed image compression is a process in which several sensor nodes with a certain computing capacity cooperate to complete the image compression task, thus reducing the workload of a single node. Collecting, compressing, coding, and transmitting numerous high resolution images in the network require substantial energy. If these tasks are concentrated on a single camera node, the used storage and processing pressure are increased, leading to instantaneous energy depletion. Through distributed image compression, the energy consumption of the network can be balanced, and the life cycle of the network can be extended [18,19,20]. However, because the networks still use the limited battery for power sources, if the depleted battery is not replaced, the network will deplete soon.

With the continuous improvement of energy harvesting technology, solar, thermal, and mechanical vibrations in the surrounding environment have been converted into available electricity to supply sensor nodes [21,22,23]. In this paper, we consider using solar energy for node energy replenishment with the sensor node’s limited battery and propose a novel distributed image compression scheme for energy harvesting WMSNs. In the proposed scheme, two level clustering, the camera node-normal node cluster and normal node cluster, is adopted. The re-clustering and dynamic adjustment methods for normal nodes are also employed for the adaptive adjustment operation mode.

The rest of this paper is organized as follows. Section 2 introduces the related works on the distributed image compression scheme for WMSNs. Section 3 presents the model assumptions. The detailed distributed image compression scheme based on solar energy harvesting is presented in Section 4. The experimental verification and analysis are presented in Section 5. Finally, conclusions are derived in Section 6.

## 2. Related Works

Considering the distributed image compression problem in WMSNs, the current research direction is mainly the optimization of the cluster structure and cluster head selection to reduce energy consumption and improve the life cycle of the network.

Lu et al. proposed a cluster structure based on JPEG2000, in which various roles were assigned to different nodes; the two hop cluster structure was adopted to transmit images [18]. Based on the role division of network nodes, Heng et al. adopted a multihop, layered routing scheme, which balances the energy consumption of each node, to transmit images [19]. Bejaoui et al. proposed a cluster based routing protocol from the perspective of network congestion, considering maximum cluster head utilization; the possible congestion in and the energy consumption of the network can be reduced, and the number of cluster heads can be minimized by balancing the number of nodes in each cluster [20]. Kumar et al. considered the difference in the residual energy of wireless multimedia sensor nodes and proposed a heterogeneous clustering scheme based on the weighted election probabilities of each node. Cluster heads were selected in accordance with the residual energy, thus improving the life cycle of the network [24]. In the clustering scheme proposed by Shiokawa et al., the residual energy of the node and the time at which the node was expected to be the cluster head were considered during cluster head node selection, thus improving transmission efficiency [25]. To improve the efficiency of Quality-of-Service (QoS) aware routing, Spachos et al. proposed an an angle based QoS and energy aware dynamic routing scheme designed for WMSNs, which optimized the selection of the forwarding candidate set and extended the network lifetime by using the inclination angle and the transmission distance between nodes [26]. Hasan et al. proposed a mathematical model for a novel QoS routing determination method. the proposed model enabled determining the optimal path to provide appropriate shared radio satisfying the QoS for a wide range of real-time intensive media [27].

The above schemes optimized cluster head node selection or QoS routing, thus improving the survival time of the network to a certain extent. However, the nodes had limited energy, which means that the network cannot work continuously for a long period, leading to network death, which causes considerable human and financial costs. Energy harvesting technologies can solve the limited life cycle of networks by providing continuous energy supply [28,29,30]. Solar energy, which can be easily obtained in the natural environment, is a good energy source due to its cleanliness, environmental friendliness, and low cost. However, several problems, such as large fluctuation of solar energy and uneven energy consumption of nodes, remain in practical applications [31,32,33]. Methods that can effectively combine an energy harvesting technology and the distributed image compression scheme are urgently needed for WMSNs.

To address this problem, we propose a distributed image compression scheme for WMSNs based on solar energy harvesting, which can effectively improve the working efficiency and stability of the network. For example, we can use this scheme in a bad environment that human beings cannot reach easily. First, we can deploy the camera nodes and put some normal nodes around them. After that, the network could be powered by solar energy supply for long periods of time without human intervention. When the normal nodes are damaged, they only need to be replenished by throwing the nodes. This would greatly reduce the frequency of network replacement, therefore decreasing the cost of network construction.

## 3. Model Assumptions

### 3.1. Topology Analysis

WMSNs have numerous sensor nodes. In consideration of the role of such nodes, the topology of WMSNs is generally divided into two types: single level planar structure and cluster based topology. From the perspective of image compression, distributed image compression requires cooperation among nodes, which is difficult to achieve through a single level plane structure. By contrast, a cluster based topology can easily realize multinode management and collaboration; thus, it is more suitable for image compression.

### 3.2. Network Model Assumptions

**Figure 1:**
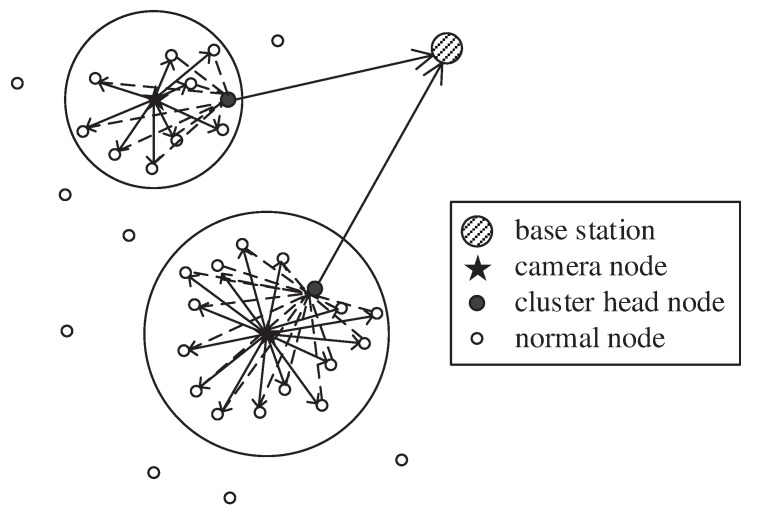
Network model.

In this section, we describe our network model assumptions, as shown in Figure 1.

- The sensor nodes in the network have two types (normal sensor nodes and nodes equipped with cameras), because equipping all nodes with image capture capability is economically infeasible and sometimes unnecessary, especially for large scale and dense WMSNs.
- In this model, the number of nodes equipped with cameras is much less than that of the normal sensor nodes, because those equipped with cameras have large communication radii.
- In this paper, the problem of image overlapping is not considered because of the sparse distribution of camera nodes; thus, the correlation of images observed by the camera nodes is small.
- The image acquisition strategy used in this paper is based on point coverage. Only the coverage of specific small areas is considered; the coverage of one line or the whole area is not considered.
- All sensor nodes are equipped with processors that can perform some highly complex processing operations.
- All nodes in the network are synchronized in time.

### 3.3. Energy Consumption Model

In WMSNs, the energy of the nodes is mainly consumed during data acquisition, data processing, and data sending and receiving.

Heinzelman’s model [34,35] was adopted for node communication consumption. The energy consumed by sending or receiving lbit data between two nodes with a distance of *d* is: $E_{tx}\left(l,d\right)$, $E_{rx}\left(l\right)$.

(1) $$E_{tx}\left(l,d\right)=\left\{\begin{array}{cc} E_{elec}\cdot l+\varepsilon_{fs}\cdot l\cdot d^{2}, & d<d_{0}\\ E_{elec}\cdot l+\varepsilon_{mp}\cdot l\cdot d^{4}, & d\geq d_{0} \end{array}\right.$$

(2) $$E_{rx}\left(l\right)=E_{elec}\cdot l$$

where $E_{elec}$ is the energy dissipated per bit, *d* is the distance between the sending node and the receiving node, $\varepsilon_{fs}$ and $\varepsilon_{mp}$ are the energy dissipated by the amplifier, which depends on the specification of the sending amplifier.

For node data processing consumption, the wavelet based image compression standard JPEG2000 was selected as the image compression method in this paper. The data processing energy consumption per bit is:

(3) $$E_{comp}=E_{DWT}\times \sum_{L=1}^{L_{0}}{\left(\frac{1}{4}\right)}^{L-1}+E_{Code}$$

where $E_{DWT}$ is the energy dissipated for one level wavelet transform per bit, $E_{Code}$ is the energy dissipated while coding, and $L_{0}$ is the wavelet decomposition level.

### 3.4. Energy Harvesting Model

In this paper, the nodes are equipped with solar panels, through which the nodes can obtain solar energy. The following assumptions are made regarding the energy supply model:

- Slight differences in radiation angle are not considered, and solar radiation intensity is considered to be the same.
- The conversion efficiency of solar panels at all nodes is fixed and equal.
- The solar panel area refers to the effective working area.

**Figure 2:**
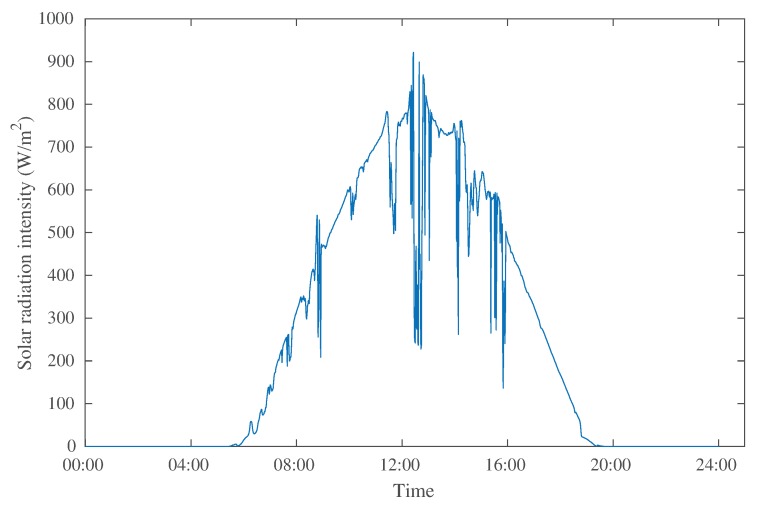
Monitoring data of solar radiation intensity.

Energy supply model:

The actual power of a solar panel with an effective area of *s* is P(s).

(4) $$P\left(s\right)=P_{s}\times s\times \eta$$

where $P_{s}$ is the actual solar radiation intensity; *s* is the effective solar panel area that receives light; η is the rated conversion efficiency of the solar panel. In our energy supply model, solar radiation intensity was derived from actual solar radiation intensity monitoring data, and η was set as 15%, which is the average efficiency of polysilicon solar panels. Figure 2 shows the solar radiation intensity monitoring data on 2 May 2018 in Oak Ridge, Tennessee, USA [36].

According to Equation (4), the energy supply obtained by the node in a picture transmission cycle *T* is $E_{get}$.

(5) $$E_{get}=P\left(s\right)\times T$$

## 4. Distributed Image Compression Scheme Based on Solar Energy Harvesting

On the basis of the above assumption, our work is to propose a novel distributed image compression scheme, which can greatly extend the survival time of the network without affecting the efficiency or even make the network survive for a long time.

The proposed scheme in this paper is based on the one proposed by Lu et al. [18], although it uses solar energy to power the wireless multimedia sensor nodes. Moreover, it adds a dynamic adjustment function to the network, which can greatly reduce the risk of sensor network death during rainy days.

The proposed scheme is cluster based, including camera node-normal node and normal node clusters. In accordance with the LEACH algorithm, the scheme is based on rounds. Each round starts from the establishment stage of the camera node-normal node cluster, followed by the establishment stage of the normal node cluster, in which the cluster head is selected by a specific algorithm. Finally, in the steady state stage, the network transmits information. From the second round onward, each round only includes the establishment stage of the normal nodes and the steady state stage.

### 4.1. Determination of Parameters

For our proposed scheme, we first need to specify some parameters, including the solar panel area and the initial energy of the node.

#### 4.1.1. Area of the Solar Panel

The solar energy supply of each node must be greater than the energy consumption under the maximum load to ensure the long term survival of the network. The maximum load and the energy collected by the solar panel can be calculated as follows:

(6) $$\left\{\begin{array}{c} E_{\max\_camera}=E_{tx}\left(D,R_{c}\right)*n\\ E_{\max\_normalNode}=\max\left\{E_{tx}\left(D,\sqrt{2}*L\right),E_{comp}\left(D\right)+E_{rx}\left(D\right)+E_{tx}\left(D,\sqrt{2}*L/N_{area}\right)\right\}\\ E_{s}=\bar{P}*S*\mu \end{array}\right.$$

where $E_{max\_camera}$ is the average energy dissipation of the camera node at the maximum load per round; $E_{max\_normalNode}$ is the average energy dissipation of the normal node at the maximum load per round; *D* is the maximum amount of picture data, which is set as 1024×1024×8b; $R_{c}$ is the camera radius; *L* is the regional side length; *n* is the number of images sent in a round; $N_{area}$ is the number of subregions (mentioned below); *P* is the mean radiation intensity; μ is the photoelectric conversion efficiency of the solar panels.

The solar panel is required to provide energy that is greater than the energy consumption of the nodes under the maximum load; thus, the inequality of the area of the solar panel *S* must be obtained.

(7) $$\left\{\begin{array}{ccc} S_{\mathrm{camera}} & \geq & \frac{E_{\max\_camera}}{\bar{P}*\mu }\\ S_{normalNode} & \geq & \frac{E_{\max\_normalNode}}{\bar{P}*\mu } \end{array}\right.$$

Based on cost considerations, we set the area of the solar panels at different nodes to meet the minimum value under inequality conditions.

#### 4.1.2. Calculate the Battery Capacity of Nodes

For the network to work immediately after construction, we used lithium batteries, which can enable the node to continue working under a temporary no-light condition. Based on the nature of the task of collecting pictures, we set the time at 30 minutes, that is 30 rounds of image transmission.

(8) $$\left\{\begin{array}{cc} E_{0_{camera}} & \geq \;30*E_{\max\_camera}\\ E_{0_{normalNode}} & \geq \;30*E_{\max\_normalNode} \end{array}\right.$$

where $E_{0\_camera}$ is the battery capacity of the camera node; $E_{0\_normalNode}$ is the battery capacity of the normal node.

As shown in Equation (8), the camera and normal nodes had different battery capacity requirements due to different values of maximum energy consumption. The camera node was mainly responsible for acquiring images and sending them to the surrounding normal nodes. The transmission distance was relatively close; thus, energy consumption was small. However, the normal node was mainly responsible for image compression. When the normal node was selected as the cluster head node, it had also to transmit the compressed image to the base station, which was far away from the normal node. Therefore, the average maximum energy consumption of the normal node was large, making the battery capacity requirement high.

### 4.2. Establishment of the Camera Node-Normal Node Cluster

Establishing the camera node-normal node cluster is the process in which the camera nodes are taken as the cluster heads, and each normal node selects a camera node cluster to merge into. Once the camera node-normal node cluster is established, it will not change; therefore, it is only performed once. This process is divided into five steps:

1. The camera node sends a broadcast with a communication radius *R*, which contains the camera node ID.
2. After receiving the broadcast from the camera node, the normal node merges into this camera node cluster. The node then sends the join broadcast containing the camera node ID and its own ID.
3. After the camera node receives the join broadcast of the normal node, the normal node ID is stored by the camera node.
4. The normal node that does not join the cluster sends a reclustering broadcast and searches for a cluster that can be joined. It continuously expands its communication radius until it receives the reclustering message sent by the camera node. When the communication radius of the normal node has covered the whole area, the broadcast stops.
5. After receiving the reclustering broadcast sent by the normal node, the camera node determines whether the normal node can join the cluster. If it can, the camera node sends the reclustering response broadcast to the normal node; otherwise, the broadcast is ignored.

During this stage, each camera node sends a broadcast at a fixed radius. The fixed radius R is determined in advance on the basis of the following principles: ensuring that each camera node does not coincide and maximizing the use of normal nodes.

**Figure 3:**
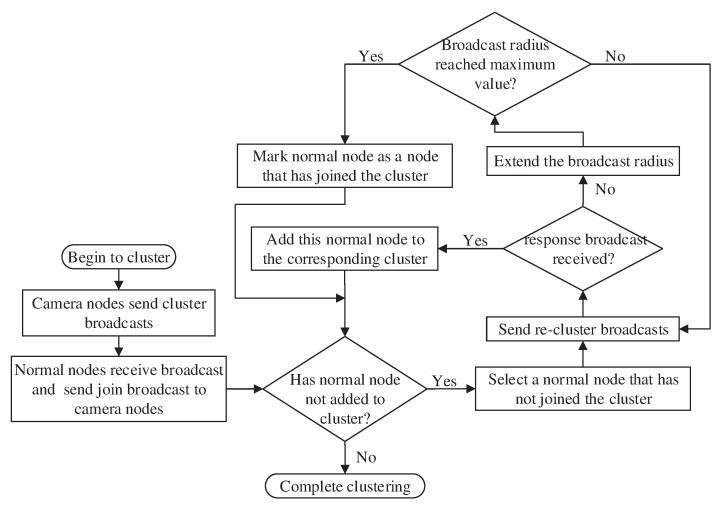
The flowchart of the camera node-normal node cluster establishment stage.

The flowchart of camera node-normal node cluster establishment stage is shown in Figure 3.

During the establishment of the camera node-normal node cluster, the concrete implementation scheme of the reclustering process must be addressed.

#### Concrete Implementation Scheme of the Reclustering Process

When cameras establish a cluster in a fixed broadcast radius, there will be a certain number of unowned normal nodes in the network, because the distance from any camera is greater than the fixed broadcast radius, and the normal node does not receive the broadcast sent by camera node and does not join any cluster. This leads to the failure of these nodes to work properly, which leads to the problem that some clusters have too few normal nodes and the waste of normal nodes. The re-clustering process can solve this problem, and the specific process is as follows:

1. Normal nodes, without joining in any clusters, send a broadcast within a radius of $\frac{d_{0}}{2}$, where $d_{0}$ is the given threshold for communication energy consumption with the free space channel model or multipath channel model. In general, $d_{0}$ = 87 m [35]. The broadcast includes its own coordinate information.
2. The camera receiving the broadcast checks the number of normal nodes in its cluster. If the number is greater than the average number of normal nodes in each cluster, the broadcast is ignored. Otherwise, the camera node sends a response broadcast to the normal node, including its distance from the node.
3. If the normal node does not receive the response broadcast, the node will become an unowned normal node. Otherwise, the normal node will compare the distance information in different response broadcasts, select the nearest camera node, and send a confirmation broadcast to it.
4. After the camera node receives the confirmation broadcast from the normal node, it adds the normal node to the member list of its own cluster. Finally, the reclustering process is completed.

### 4.3. Establishment Stage of the Normal Node Cluster

In this stage, we first select the cluster head node using a specific algorithm and then merge all normal nodes into the nearest cluster.

The algorithm for cluster head node selection proceeds as follows:

1. The region for detection is divided into Nareaparts (the value of Narea depends on the size of the region).
2. In each region, the node (other than the camera node) closest to the base station with a residual energy greater than the threshold value is selected as the cluster head node.

**Figure 4:**
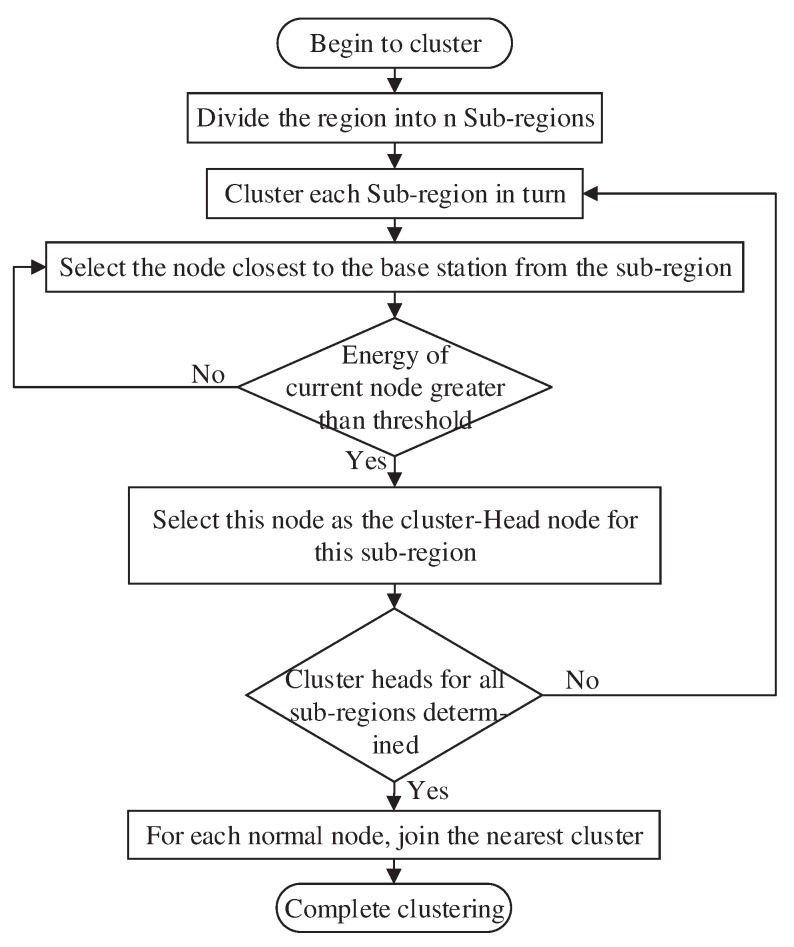
Normal node cluster establishment stage.

The flowchart of the establishment of the normal node cluster is shown in Figure 4.

Three issues must be addressed in this stage: how to set the energy threshold in the cluster head selection algorithm, how to determine the number of subregions $N_{area}$ and how to divide the region after the number of subregions $N_{area}$ has been determined.

#### 4.3.1. Setting the Energy Threshold

Simulation showed that the energy consumption of the cluster head node was much higher than that of the normal node in the steady state. Therefore, nodes with more energy should be selected as cluster head nodes. The energy threshold was set to exclude some nodes with less energy, thus ensuring that the cluster head node would not die due to energy consumption in the following steady state. Each round was only one minute apart; therefore, the solar energy collected in this round was predicted to be equal to the solar energy collected in the previous round.

In summary, the basic value of the threshold was set as the energy consumed by the cluster head node in each round under steady state $E_{H}$.

(9) $$\left\{\begin{array}{cc} E_{H} & =\;E_{tx_{H}}+E_{rx_{H}}-E_{get}\\ E_{tx_{H}} & =\;E_{tx}\left(\frac{n\times M\times N\times 8}{r\cdot clusterHeadNum},{d_{CH}}_{-BS}\right)\\ E_{rx_{H}} & =\;E_{rx}\left(\frac{n\times M\times N\times 8}{r\cdot clusterHeadNum}\right)\\ E_{get_{cur}} & =\;E_{get_{last}} \end{array}\right.$$

where $E_{tx_{H}}$ is the energy that the cluster head node consumes to send pictures in one round; $E_{rx_{H}}$ is the energy that the cluster head node consumes to receive pictures in one round; $E_{get}$ is the predicted solar energy that a round can collect; $E_{get_{last}}$ is the actual solar energy collected in the previous round; clusterHeadNum is the number of cluster head nodes; *n* is the number of pictures sent in one round; $d_{CH-BS}$ is the distance from the cluster head node to the base station; *M* and *N* are the image pixel size.

To prevent normal nodes from dying after becoming a cluster head node (that is, it still had energy to perform image compression), we added a value to the threshold. The added value was the energy Es that would be consumed as a normal node in one round.

(10) $$\left\{\begin{array}{cc} E_{s} & =\;E_{tx_{s}}+E_{rx_{s}}+E_{\mathrm{com}p_{s}}\\ E_{tx_{s}} & =\;E_{tx}\left(\frac{n\times M\times N\times 8}{r\times normalNodeNum},d_{per}\right)\\ d_{per} & =\;\bar{\sum dist(i,j)},i\in V_{H},j\in V_{s}\\ E_{rx_{s}} & =\;E_{rx}\left(\frac{n\times M\times N\times 8}{normalNodeNum}\right)\\ E_{comp_{s}} & =\;\frac{E_{comp}\times n\times M\times N\times 8}{normalNodeNum} \end{array}\right.$$

where $E_{tx_{s}}$ is the energy dissipation by a normal node sending pictures in one round; $E_{rx_{s}}$ is the energy dissipation by a normal node receiving pictures in one round; $E_{comp_{s}}$ is the energy dissipation by a normal node compressing pictures in one round; normalNodeNum is the number of normal nodes; $d_{per}$ is the average distance between normal nodes and cluster head nodes; dist(i,j) is the distance between node *i* and node *j*; $V_{H}$ is the set of cluster head nodes; $V_{s}$ is the set of normal nodes.

#### 4.3.2. Determining the Number of Subregions

We divided the entire network region into equal subregions, and each subregion was a monitoring area. A camera node was set in each subregion, and a cluster head node was selected from the normal nodes in the latter stage. Each subregion needed enough normal nodes to perform image rotation compression. However, a large subregion would increase the energy dissipation of image transmission within the region. Therefore, we provide the following inequality:

(11) $$\left\{\begin{array}{c} N_{comp}\;=\;\frac{T_{round}\times F_{camera}}{normalNodeNum\times \frac{1}{Narea}}\;\leq \;1\\ \frac{S_{area}}{N_{area}}\;\leq \;{d_{0}}^{2} \end{array}\right.$$

where $N_{comp}$ is the average number of compressed images per normal node in a round; $T_{round}$ is the time consumed by one round; $F_{camera}$ is the image transmission frequency of the camera node; $N_{area}$ is the number of sub-regions; $S_{area}$ is the area of the network region.

#### 4.3.3. Specific Division of the Region

After calculating the number of subregions in the last section, we must divide the region. Considering that the communication areas of nodes were all round, we set the division area as square as possible. We divided the region proportionally into *m* rows and *n* columns.

(12) $$\left\{\begin{array}{c} m_{d}\;\leq \;\sqrt{N_{comp}}\\ m_{d}\;\mathrm{is}\;\mathrm{the}\;\mathrm{factor}\;\mathrm{of}\;N_{comp}\\ n_{d}\;=\;\frac{N_{comp}}{m_{d}} \end{array}\right.$$

### 4.4. The Steady State Phase

In this phase, the camera node sets the sending frequency and picture quality according to the specific working mode and sends the picture to the normal node. After receiving images, the normal nodes determine the compression rates according to the residual energy of their cluster head nodes and send the compressed images to the cluster head nodes. The cluster head nodes then send images to the base station.

**Figure 5:**
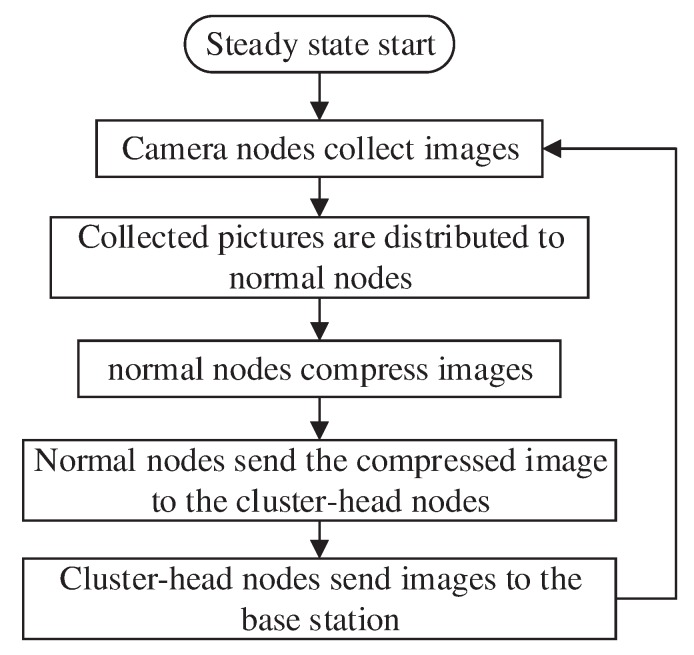
The flowchart of the steady state phase.

The flowchart of the steady state phase is shown in Figure 5.

At this stage, we mainly address three issues: the initial working mode setting of the camera node, the cooperative adjustment algorithm between the camera and normal nodes, and the determination of the maximum compression rate Rcomp_Max.

#### 4.4.1. Initial Working Mode Setting of the Camera Node

Working mode refers to the set of controllable factors that affect the working condition of nodes. For camera nodes, controllable factors include image acquisition frequency and resolution. Changing the working mode of the camera node can adjust its workload, thus affecting its energy consumption. The initial working mode of the camera node is the working condition when the node starts to work after completing network construction.

In this scheme, the initial working mode of the camera node is set as the working condition of the camera node under the highest workload. To balance the energy consumption of the network and reduce the working pressure of the cluster head nodes around the camera nodes far away from the base station, this scheme divides the workload into three grades according to distance d between the camera node and the base station. The specific division scheme is as follows:

(13) $$\left\{\begin{array}{ccc} M=N=1024, & n=60, & 0<d\leq \frac{d_{0}}{2}\\ M=N=512,\;\; & n=60, & \frac{d_{0}}{2}<d\leq d_{0}\\ M=N=512, & n=30, & d_{0}<d\leq L \end{array}\right.$$

#### 4.4.2. Cooperative Adjustment between Nodes

Nodes indirectly adjust to other nodes in the same working chain by adjusting their own working mode, aiming to ensure the survival of nodes in the network and to balance network energy dissipation. This scheme mainly proposes two collaborative adjustment methods.

(1) Cooperative adjustment between normal nodes and camera nodes: When the power of an ordinary node is insufficient, the adjustment broadcast is sent to the camera node. The camera node adjusts its own image acquisition frequency and resolution according to the energy condition of the normal node and its own energy condition, thus indirectly adjusting the workload of the normal node. The specific adjustment algorithm is shown in Algorithm 1 and Algorithm 2.
**Algorithm 1:** Autonomous adjustment algorithm for camera nodes.**Input**: $C_{i}$, the camera node in a cluster; $E_{c}$, the energy of $C_{i}$; $E_{r}$, the ratio of the solar energy to the energy consumed by $C_{i}$ each round.**Output**: n, the number of images for $C_{i}$ to send.**Step**: 11)$E_{n},E_{r}=C_{i}.getEnergyInfo\left(\right);$//(Fetch the energy info of $C_{i}$(2)**while**$E_{c}>0$**do**//($C_{i}$ has energy((3)  **if**
$E_{c}<\frac{E_{0}}{2}\;\&\;E_{r}<1$
**then**//($C_{i}$ lacks energy((4)   $n=n\times \lceil E_{r}\rceil ;$//(Adjust the number of images(5)  **else if**
$E_{c}\geq \frac{E_{0}}{2}\;\&\;E_{r}\geq 1$
**then**//($C_{i}$ has enough energy((6)   $M,n=C_{i}.initialize\left(\right);$7)  **endif**(8)**endwhile**(9)**return***n*;**Step**: 210)$E=C_{i}.calEnergy\left(M,N\right);$//(E is the energy for sending images(11)**if**$E>E_{c}$**then**(12)  $C_{i}.skipRound\left(\right);$13)**else**(14)  $C_{i}.sendImage\left(\right);$15)**endif**(

**Algorithm 2:** Cooperative adjustment algorithm between normal nodes and camera nodes.**Input**: $C_{i}$, the camera node in a cluster; $N_{i}$ is one of the normal nodes that belongs to $C_{i}$; $E_{c}$ is the energy of $C_{i}$; $E_{n}$ is the energy of $N_{i}$; $E_{r}$ is the ratio of the solar energy to the energy consumed by $N_{i}$ each round.**Output**: The signal type ($signal\_0:M=N=512,n=\lceil \frac{n}{2}\rceil$; $signal\_1:C_{i}.initialize\left(\right)$)**Step**: 11)
$E_{n},E_{r}=N_{i}.getEnergyInfo\left(\right);$

//
(Fetch the energy info of $N_{i}$(
2)
**while**
$E_{c}>0\;\&\;E_{n}>0$
**do**

//
($N_{i},C_{i}$ has energy(
(
3)  **if**
$E_{n}<\frac{E_{0}}{2}\;\&\;E_{r}<1$
**then**
//
($N_{i}$ lacks energy(
(
4)   **return**
signal_0;
//
($N_{i}$ sends signal_0 to $C_{i}$(
5)  **else if**
$E_{n}\geq \frac{E_{0}}{2}\;\;\&\;\;E_{r}\geq 1$
**then**
//
($N_{i}$ has enough energy(
(
6)   **return**
signal_1;
//
($N_{i}$ send signal_0 to $C_{i}$(
7)  **endif**(8)
**endwhile**
(
**Step**: 29)$Signal=C_{i}.getSignal\left(\right)$;10)
**if**
Signal==signal_0
**then**
(
11) $M=N=512,\;n=\lceil \frac{n}{2}\rceil$;
//
($C_{i}$ adjusts its working mode(
12)
**endif**
(
13)
**if**
Signal==signal_1
**then**
(
14)  $C_{i}.initialize\left(\right)$;
//
($C_{i}$ initializes its working mode(
15)
**endif**
(
16)$E=C_{i}.calEnergy\left(M,n\right)$;
//
(E is the energy for sending images(
17)
**if**
$E>E_{c}$
**then**
(
18)  $C_{i}.skipRound\left(\right)$;19)
**else**
(
20)  $C_{i}.sendImage\left(\right)$;21)
**endif**
(


(2) Cooperative adjustment algorithm between normal nodes and cluster head nodes: When the remaining energy of the cluster head node is insufficient, the adjustment broadcast and its own energy information are sent to all the normal nodes in the cluster. According to the energy information of the cluster head node and its own energy, the normal node adjusts its own image compression rate, thus indirectly adjusting the workload of the cluster head node. The specific adjustment algorithm is shown in Algorithm 3.

#### 4.4.3. Determination of the Maximum Compression Rate Rcomp_Max

The PSNR of image restoration is required to be greater than 30 dB to ensure the quality of the final collected images. According to the relationship between the compression rate and PSNR in Standard Test Images 1, we adopted 0.25 bpp, that is the compression rate $R_{comp\_max}$ was $\frac{1}{32}$ under the 8 bit image.

#### 4.4.4. Determination of the Maximum Compression Rate Rcomp_Max

The PSNR of image restoration is required to be greater than 30 dB to ensure the quality of the final collected images. According to the relationship between the compression rate and PSNR in Standard Test Images 1, we adopted 0.25 bpp, that is the compression rate $R_{comp\_max}$ was $\frac{1}{32}$ under the 8 bit image.
**Algorithm 3:** The cooperative adjustment algorithm of normal nodes and cluster head nodes.**Input**: $C_{i}$, the camera node in a cluster; $N_{i}$, one of the normal nodes that belongs to $C_{i}$; $H_{h}$, the cluster head node of this cluster; $E_{c}$, the energy of $C_{i}$; $E_{h}$, the energy of $C_{i}$; $E_{r}$, the ratio of the solar energy to the energy consumed by each round.**Output**: The signal type (signal_0, adjust compression rate; signal_1, initialize compression rate)**Step**: 11)$E_{h},E_{r}=N_{h}.getEnergyInfo\left(\right);$//(Fetch the energy info of $N_{h}$(2)**while***$E_{c}>0\;\&\;E_{h}>0\;\&\;E_{n}>0$***do**//($C_{i},N_{i},H_{h}$ has energy((3)  **if**
$E_{h}<E_{0}\;\&\;E_{r}<1$
**then**//($N_{h}$ lacks energy((4)   **return**
signal_0;//($N_{i}$ sends signal_0 to camera(5)  **else if**
$E_{n}\geq \frac{E_{0}}{2}\;\&\;E_{r}\geq 1$
**then**//($N_{h}$ has enough energy((6)   **return**
signal_1;7)  **endif**
(8)**endwhile**(**Step**: 29)$Signal=N_{i}.getSignal\left(\right);$;10)**if**Signal==signal_0**then**(11)  $r=r_{i}\;\left(i=1,2,3,4\right)$;//(Adjust compression rate(12)**endif**(13)**if**Signal==signal_1**then**(14)  $N_{i}.initialize\left(\right);$//(Initialize compression rate(15)**endif**(16)$E=N_{i}.calEnergy\left(M,N,r\right);$//(E is the energy for compressing images(17)**if**$E>E_{r}$**then**(18)  $N_{i}.skipRound\left(\right);$;19)**else**(20)  $N_{i}.compressImage\left(\right);$;21)**endif**(

## 5. Performance Evaluation

In this section, we evaluate the performance of the proposed distributed image compression scheme using MATLAB. In consideration of the random distribution of 100 nodes in a 100 m × 100 m rectangular region, the base station was located at (0,0), and six camera nodes $C_{1}$∼$C_{6}$ were uniformly distributed in the region to collect pictures periodically.

**Figure 6:**
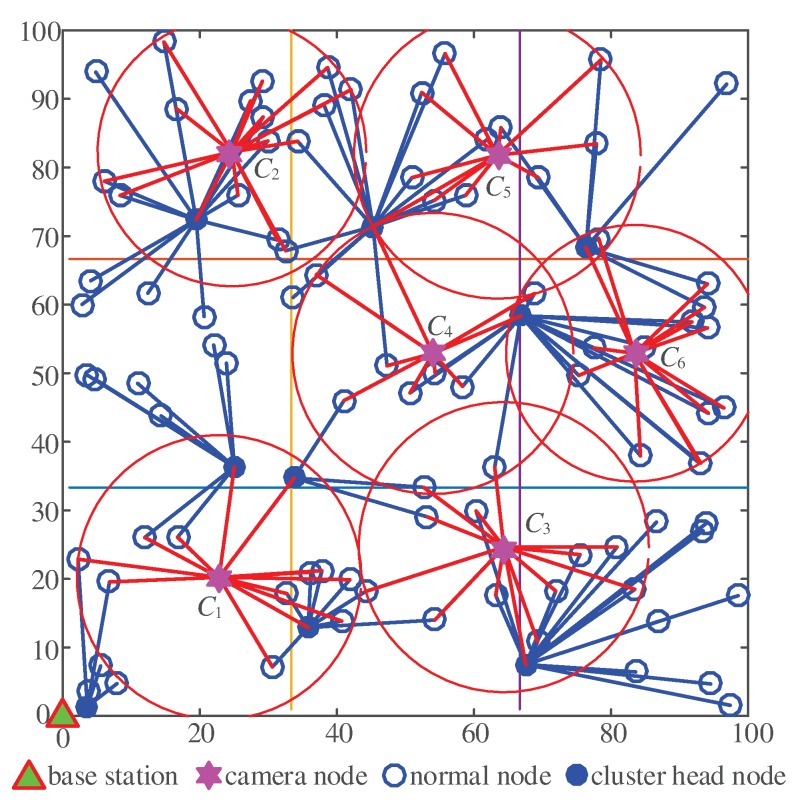
Distribution of the camera nodes.

The six camera nodes were located at (20,20), (20,80), (60,20), (60,80), (50,50), (80,50), and (80,50). The coordinates were fluctuated up and down by 5 m, as shown in Figure 6. This simulated throwing the nodes to the specific locations, and the fluctuation simulated the error during throwing. The initial work modes of the camera nodes were set according to the distances from the base station as described above.

The values of the energy model parameters were chosen as follows: the values of the parameters of the wireless communication energy model in Equations (1) and (2) were the typical values $E_{elec}$ = 50 nJ/bit, $\varepsilon_{fs}$ = 10 pJ/bit/m${}^{2}$, $\varepsilon_{mp}$ = 0.0013 pJ/bit/m${}^{4}$, and $d_{0}$ = 87 m. In our image compression scheme, the energy dissipation of the sending node and the receiving node would be calculated according to Equations (1) and (2). For the node data processing consumption in Equation (3), $E_{DWT}$ was set as 220 nJ/bit, whereas $E_{Code}$ was set as 20 nJ/bit [18]. $B_{r}=0.25$ bpp. The initial energy of the camera node was 802.2 J, and that of the normal node was 1547 J.

We equipped the nodes with solar panels. The solar panels of the normal nodes were 14 cm × 14 cm, and those of the camera nodes were 10 cm × 10 cm. The cluster head nodes would be changed in each round, and the number of normal nodes was large; thus, we considered the death of camera nodes (the remaining energy was not enough for the next round of image transmission and collection) as the end of the network life. Our simulation used solar radiation intensity monitoring data from 12:00 pm on 1 May to 12:00 pm on 2 May, 2018 in Oak Ridge, Tennessee, USA [36]. In these datasets, there was no solar radiation approximately from Rounds 500 to 1000, because the period of time was at night without sunlight.

### 5.1. Network Performance Evaluation

In this section, we observe the changes of various parameters of the network with the changes of solar radiation intensity to analyze the performance of the proposed distributed image compression scheme.

#### 5.1.1. Image Received by the Base Station

**Figure 7:**
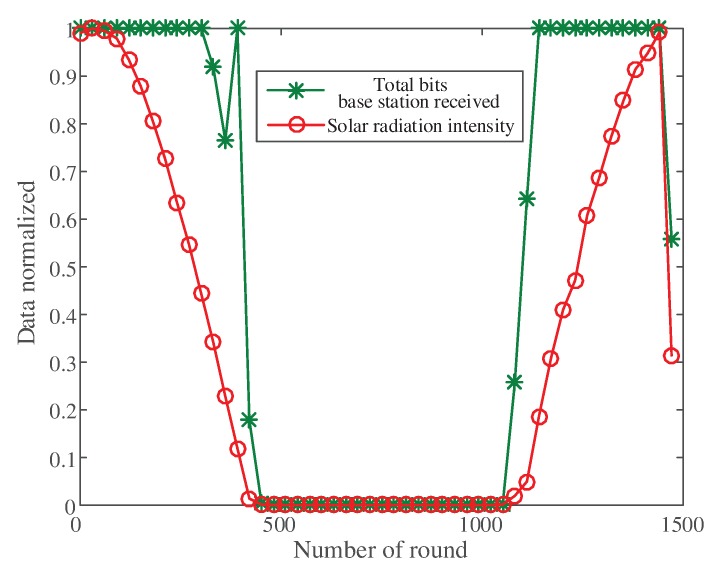
The curve of the solar radiation intensity and the sum of the number of image bits received by the base station.

Figure 7 shows the relationship between the image received by the base station (the sum of the number of bits) and the solar radiation monitoring intensity in each round. When the solar radiation intensity was strong, the transmission efficiency of the network and the number of bits received by the base station was high. When the solar radiation intensity decreased, the sum of the number of bits was initially unchanged. When the remaining energy of nodes became insufficient, the sum of the number of bits received by the base station decreased due to dynamic adjustment. The dynamic regulation function was based on the residual energy of the nodes. Therefore, when the solar radiation intensity started to decrease, the residual energy of the nodes was sufficient and dynamic adjustment did not trigger. That was why the sum of the number of bits was unchanged. The received bits increased with solar radiation rising.

#### 5.1.2. Residual Energy of the Camera Node

In the network, the camera node was mainly responsible for image collection. When the camera nodes died due to insufficient energy, we considered the network dead. Therefore, the residual energy of the camera node was an important indicator for network performance evaluation.

**Figure 8:**
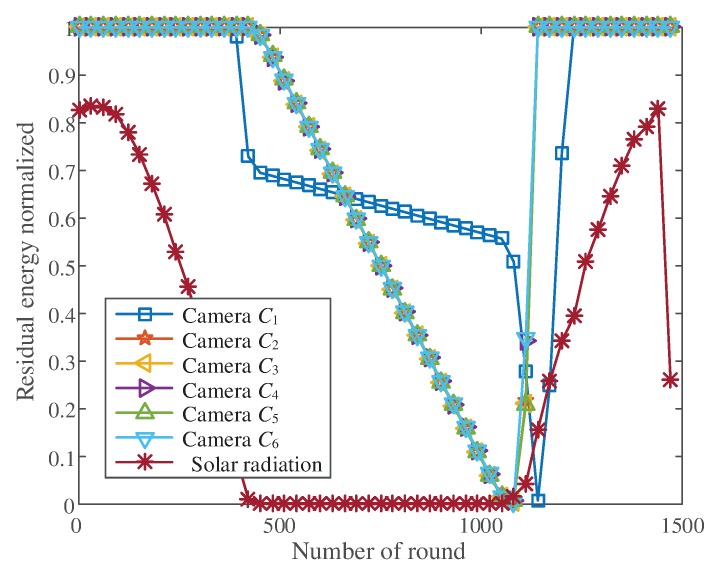
Residual energy change curve of camera nodes.

The remaining energy of each round of the camera node is shown in Figure 8. When the solar radiation intensity declined, the remaining energy of camera nodes was unaffected by the solar radiation intensity because it took less energy than solar energy supply to collect images. However, when the solar radiation intensity remained low, the remaining energy of the camera nodes decreased.

The remaining energy of the camera node close to the base station decreased first, because this camera node collected high definition images at a high frequency. The residual energy of the camera node increased with the solar radiation intensity.

### 5.2. Network Performance Comparison

In this section, we evaluate the performance of our scheme by simulating different schemes.

#### 5.2.1. Network Transmission Efficiency

We used the sum of image bits received by the base station in each round degree to evaluate the image transmission efficiency of the network. The larger the number of bits, the more efficient the network was. We compared the transmission efficiency of the following schemes:

Scheme 1: The proposed scheme, a distributed image compression scheme with solar energy supply and dynamic adjustment.

Scheme 2: An image transmission scheme based on a two hop cluster [18].

Scheme 3: A two hop cluster image transmission scheme with solar energy replenishment.

**Figure 9:**
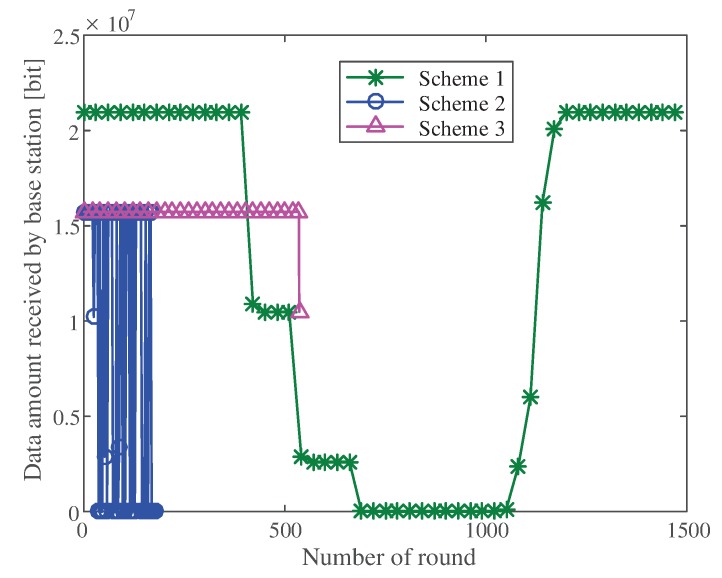
Comparison of the sum of the bits in three schemes.

Figure 9 shows the sum of the number of bits received by the base station in each round of the three schemes. When the energy was sufficient, the proposed scheme sent high definition images at a high frequency; thus, the sum of bits received by the base station was relatively high. When the remaining energy of the network was insufficient, the scheme gradually reduced the transmission frequency and clarity of the image, thus reducing the energy consumption of the network. The number of bits received by the base station was reduced accordingly. After simulation, the proposed scheme could ensure the long term survival of the network. Only a part of the data was intercepted in the figure.

Simulation results of the two hop cluster scheme are also presented. In the figure, the condition of base station not receiving images occurred in some rounds, because in the two hop cluster scheme, the energy of the cluster head node was not considered when determining the radius of the camera node. Therefore, in some cases, the cluster head node could not send images, resulting in the failure of receiving images at the base station.

The condition of the base station not receiving images in the two hop cluster scheme was addressed after adding an energy supply. However, when the solar power supply weakened, the network died because of the lack of adjustment.

It can be seen from the figure that solar energy supply could not effectively extend the survival time of the network (Scheme 3), because there was still a period of weak solar energy supply in a day. During this period, the network must be dynamically adjusted to reduce energy consumption so that the network can survive for a long time.

#### 5.2.2. The Balance Degree of Network Energy Dissipation

We set the initial energy for each node. The balance degree of network energy dissipation can be evaluated by observing the residual energy of each node after each round. The definition of the residual energy balance of nodes $R_{comp}$ is provided. Through $R_{comp}$, we can compare the energy dissipation balance degree of different schemes numerically. The greater the degree of balance, the more unbalanced the energy consumption of the network.

(14) $$R_{comp}=\frac{E_{max}-E_{min}}{E_{max}}\times 100\%$$

where $E_{max}$ is the maximum residual energy of all nodes in one round; Eminis the minimum residual energy of all nodes in one round.

**Figure 10:**
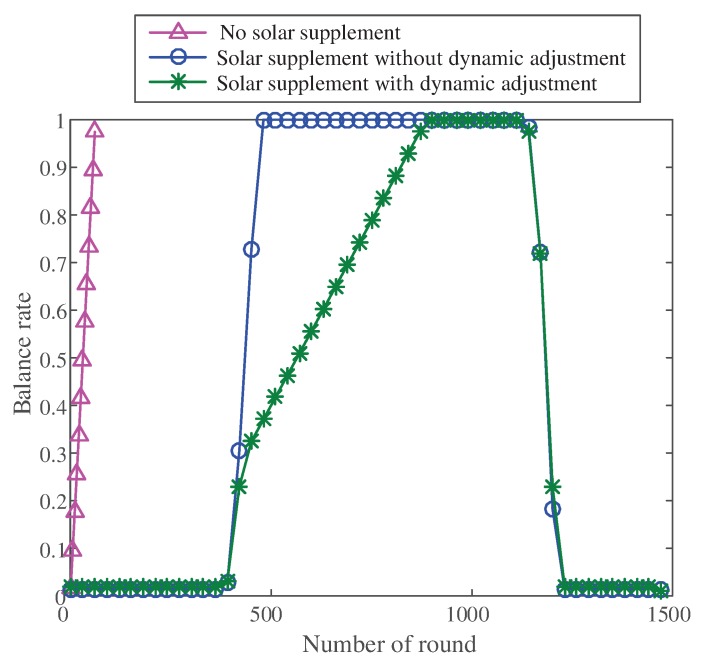
The comparison of balance.

We compared the balance of three schemes: scheme without solar supply, scheme with solar supply but without dynamic adjustment, and scheme with solar supply and dynamic adjustment. The results are shown in Figure 10.

When no solar energy supply and dynamic adjustment were available in the network, the residual energy of the network nodes was highly unbalanced, and the balance degree rose in a straight line and finally tended to a straight line. The reason for this tendency was that the normal nodes in the network lacked energy, and the camera nodes stopped sending pictures. If the network did not have dynamic adjustment and when the solar energy supply was strong, the balance degree of change was small and the residual energy of the node was balanced. However, when the solar energy supply was low, the balance degree of the network rose sharply, and the remaining energy of the nodes became highly unbalanced. When the solar energy supply recovered, the balance degree was reduced. Compared with the network without dynamic adjustment, the balance of the network with dynamic adjustment increased slowly. As shown in the figure, the rising slope of the network balance degree with dynamic adjustment changed twice. The first change occurred because the solar energy supply and the remaining energy of the nodes decreased. The second change occurred because the residual energy of the nodes in the network dropped to a certain extent, which triggered the dynamic adjustment of the network.

Figure 10 shows that by adding solar energy supply and dynamic adjustment to the network, the balance of residual energy could be well optimized, making the energy consumption of the network even.

#### 5.2.3. Network Life Cycle Evaluation

The proposed scheme could make the network survive for a long time due to solar energy supply and dynamic adjustment. To measure the life cycle of a network, we set a limit of 10 days. If a network could survive for 10 days, we believed it could survive for a long time. If a network died within 10 days, its survival time was taken as the evaluation result of its life cycle.

The solar radiation intensity data in the simulation were based on monitoring data from 1 to 10 May 2018 in Oak Ridge, Tennessee, USA. Through simulation, we found that our scheme could survive for more than 10 days. However, in the scheme with a two hop cluster without energy supply, the network died due to the energy exhaustion of camera nodes after seven hours. When solar energy was supplied to the two hop cluster scheme, the network ran for approximately nine hours and finally died due to insufficient energy of the camera node. When the solar energy supply was sufficient during daytime, the nodes of the two hop cluster network with solar energy supply had enough residual energy; however, as the solar energy supply gradually weakened, the residual energy decreased, and the network died eventually.

## 6. Conclusions

The combination of WSNs and edge computing not only enhances their capabilities, but also motivates a series of new applications. As a typical application, WMSNs have become a hot research issue. Based on the characteristics of wireless multimedia sensor energy collection, this paper adopted the distributed image compression scheme based on solar energy collection to compress and transmit the image, set different roles for different nodes, divided the image compression and transmission work, and added solar energy supply and dynamic adjustment between nodes. The simulation results showed that the distributed image compression scheme based on solar energy collection could balance the network energy dissipation, making the network survive for a long time, maximizing the performance of the network, and improving the quality and frequency of image transmission on the premise of ensuring the survival of the network. Compared with the two hop cluster scheme, our scheme could make the network survive longer, and from the perspective of solar energy supply, our scheme was more suitable for that because of the dynamic adjustment.
